# Targeting Nuclear Exporter Protein XPO1/CRM1 in Gastric Cancer

**DOI:** 10.3390/ijms20194826

**Published:** 2019-09-28

**Authors:** Rachel Sexton, Zaid Mahdi, Rahman Chaudhury, Rafic Beydoun, Amro Aboukameel, Husain Y. Khan, Erkan Baloglu, William Senapedis, Yosef Landesman, Anteneh Tesfaye, Steve Kim, Philip A. Philip, Asfar S. Azmi

**Affiliations:** 1 Department of Oncology, Wayne State University School of Medicine, Detroit, MI 48201, USA; sextonr@karmanos.org (R.S.); kameelo@karmanos.org (A.A.); khanh@karmanos.org (H.Y.K.); tesfayea@karmanos.org (A.T.); kims@karmanos.org (S.K.); Philipp@karmanos.org (P.A.P.); 2 Emory Winship Cancer Institute, Atlanta, GA 30322, USA; zaid.mahdi@emory.edu; 3 Detroit Medical Center, Wayne State University, Detroit, MI 48201, USA; arahman84@gmail.com; 4 Department of Pathology, Wayne State University School of Medicine, Detroit, MI 48201, USA; rbeydou@med.wayne.edu; 5 Karyopharm Therapeutics Inc., Newton, MA 02459, USA; ebaloglu@restorbio.com (E.B.); William@karyopharm.com (W.S.); yosef@karyopharm.com (Y.L.)

**Keywords:** gastric cancer, nuclear protein transport, XPO1/CRM1, SINE, miRNA

## Abstract

Gastric cancer remains an unmet clinical problem in urgent need of newer and effective treatments. Here we show that the nuclear export protein, Exportin 1 (XPO1, chromosome region maintenance 1 or CRM1), is a promising molecular target in gastric cancer. We demonstrate significant overexpression of XPO1 in a cohort of histologically diverse gastric cancer patients with primary and metastatic disease. XPO1 RNA interference suppressed gastric cancer cell growth. Anti-tumor activity was observed with specific inhibitor of nuclear export (SINE) compounds (selinexor/XPOVIO), second-generation compound KPT-8602/eltanexor, KPT-185 and +ve control Leptomycin B in three distinct gastric cancer cell lines. SINE compounds inhibited gastric cancer cell proliferation, disrupted spheroid formation, induced apoptosis and halted cell cycle progression at the G1/S phase. Anti-tumor activity was concurrent with nuclear retention of tumor suppressor proteins and inhibition of colony formation. In combination studies, SINE compounds enhanced the efficacy of nab-paclitaxel in vitro and in vivo. More significantly, using non-coding RNA sequencing studies, we demonstrate for the first time that SINE compounds can alter the expression of non-coding RNAs (microRNAs and piwiRNAs). SINE treatment caused statistically significant downregulation of oncogenic miR-33b-3p in two distinct cell lines. These studies demonstrate the therapeutic significance of XPO1 in gastric cancer that warrants further clinical investigation.

## 1. Introduction

Gastric adenocarcinoma is the leading cause of cancer-related deaths worldwide with an estimated 1 million newly diagnosed cases and 782,000 associated deaths annually [1]. Frequently detected in its metastatic stages due to symptoms mimicking bouts of gastrointestinal discomfort, bloating and nausea, this disease remains chemoresistant and deadly. Lack of early detection and poor response to available chemotherapeutics keeps the five-year survival rate of gastric cancer at a dismal 25% [2,3]. The histology of gastric adenocarcinomas varies among different demographics. Abiding by the Lauren classification, the intestinal subtype predominates in the United States [4]. This subtype originates from the cardia and is similar in presentation and disease progression to esophageal cancer [5]. Causes include *Helicobacter pylori* infection and older male patient populations are at highest risk with poorer survival rates [4,5]. In South America, the Middle East and Asia, the diffuse subtype predominates. It is non-cardia originating, occurs in a younger population equally effecting males and females and is more frequently associated with a heredity component [6,7]. Irrespective of the sub-type, gastric cancer requires identification of newer molecularly driven targets in order to positively impact the overall survival statistics.

The nuclear export protein, Exportin 1 (XPO1) regulates the shuttling of genome surveillance proteins, tumor suppressors (TSPs), select miRNAs and mRNAs from the nucleus to the cytosol [8]. This occurs through binding of XPO1 to a leucine-rich nuclear export sequence (NES) on the target TSPs or RNA that are facilitated by RAN-related Nuclear protein Guanine Exchange Factors (RAN-GEFs). The XPO1-cargo protein complex travels through the nuclear pore and is released in the cytoplasm upon hydrolysis of GTP-binding nuclear protein RanGTP (RanGTP) to GTP-binding nuclear protein Ran GDP (Ran-GDP) [8,9]. Many tumor types overexpress XPO1 compared to normal tissue [10,11,12]. Such hyperactivation of XPO1 results in increased activity of cargo expulsion to the cytosol largely leading to inactivation of TSPs and disrupted maturation of miRNAs and even mRNA translation [10,11,12]. Supporting these observations, hyperactivation of XPO1 enhances therapy resistance and poor overall survival in patients with solid tumors and hematological malignancies [13,14,15,16,17]. Studies from our group and others have established the utility of targeting XPO1 in several tumor types using selective inhibitors of nuclear export (SINE) compounds [13,14,15,16,17,18]. These studies led to a number of Phase I/II/III selinexor clinical trials [19,20,21,22] and selinexor (XPOVIO^TM^) received Food and Drug Administration (FDA) approval for penta-refractory multiple myeloma in 2019 [23].

In this report, we demonstrate the critical role of XPO1 in gastric cancer sustenance using diverse sub-types of patient tissues, in in vitro cellular models, 3D models as well as an in vivo xenograft model. We demonstrate that targeted inhibition of XPO1 by SINE compounds could effectively inhibit gastric tumor growth. We also unveil novel non-coding RNA targets of SINE compounds. These studies support the hypothesis that targeting XPO1 could be a viable therapeutic strategy in this recalcitrant malignancy.

## 2. Results

### 2.1. XPO1 Is Elevated in Gastric Adenocarcinoma Compared to Normal Tissues

**Figure 1:**
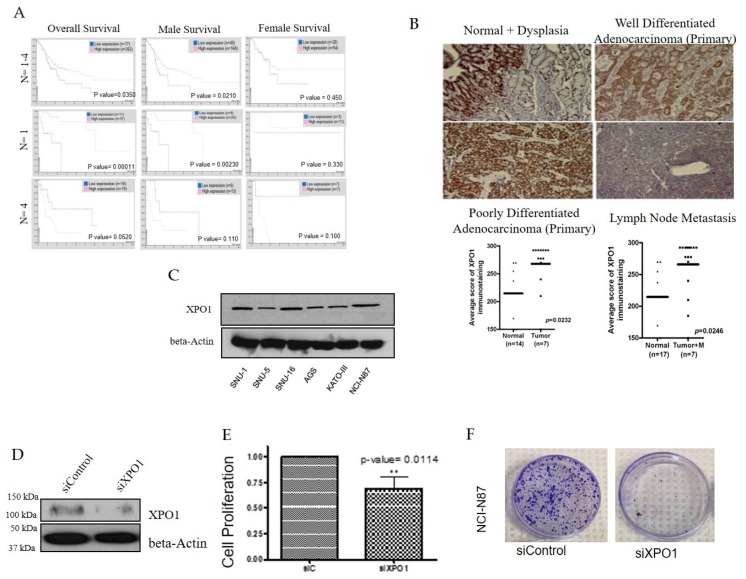
Exportin 1 (XPO1) is an essential protein in gastric cancer. (**A**) Left column: overall survival rate of gastric cancer patients with XPO1 expression levels at various stages of disease. Middle column: survival of gastric cancer patients (male) with XPO1 expression levels at various stages of disease. Right column: survival of gastric cancer patients (female) with XPO1 expression levels at various stages of disease. (**B**) A panel of gastric cancer tissues from different disease stages were stained for XPO1 expression levels using immunohistochemistry. Graph quantification of scoring is provided below. (**C**) Western blot of a panel of gastric cancer cell lines probed for XPO1 and beta-actin. (**D**) Western blot of XPO1 silenced NCI-N87 cells and beta-actin. (**E**) Growth analysis of silenced XPO1 in NCI-N87 cells (*n* = 3). ** *p* < 0.01. (**F**) Colony formation assay NCI-N87 cells (*n* = 3) post XPO1 siRNA.

Using the Human Protein Atlas, we found that high XPO1 expression is linked to survival outcomes in all stages of gastric cancer (*p* < 0.05) (Figure 1A). Since gastric cancer affects both genders, we wanted to know whether there are survival differences that correlate with XPO1 overexpression. We found that males showed a better survival rate which correlated with a high expression of XPO1 (*p* < 0.05) (Figure 1A). We also wanted to identify if the survival at a particular disease state correlated with XPO1 expression. At primary grade lesions, where (*n* = 1), high XPO1 correlated with increased overall survival and when stratified to gender, the male population had a significantly increased survival benefit (*p* < 0.05) (Figure 1A). Low XPO1 overall is more favorable for metastatic gastric cancer (*p* > 0.05), and there is no correlation to XPO1 expression between genders (Figure 1A) [24]. We further observed increased XPO1 expression in dysplastic gastric cells with low levels of dysplasia as well as invasive adenocarcinoma compared to benign normal gastric mucosa. The level of expression in metastatic lymph nodes was positive. We next evaluated the expression levels of XPO1 in a panel of gastric cancer cell lines. NCI-N87 (metastatic intestinal), SNU-1 (primary diffuse) and SNU-16 (metastatic diffuse) were chosen for further evaluation as they are all histologically diverse and express similar levels of XPO1 (Figure 1C). Having established that XPO1 is overexpressed in gastric cancer tissue, we then evaluated the impact of knocking down this protein on cellular survival. siXPO1 (Figure 1D), retarded cellular growth significantly (*p* < 0.05) (Figure 1E) and decreased colony formation (Figure 1F) compared to the control. Together, these results suggest that XPO1 inhibition of XPO1 can significantly reduce gastric cancer cell growth and could be a viable therapeutic target for this disease. These results demand an in depth pre-clinical investigation of relevant and available XPO1 inhibitors.

### 2.2. Selective Inhibitors of Nuclear Export (SINE) Target XPO1 and Inhibit Cell Viability

**Figure 2:**
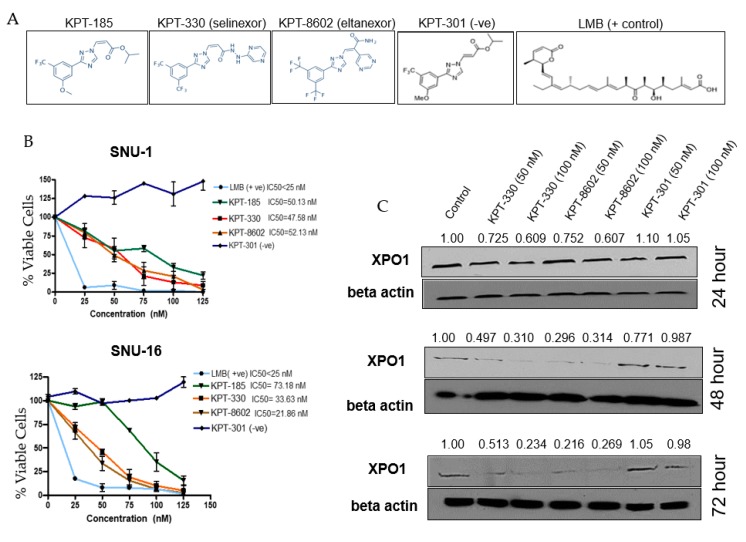
Selective inhibitor of nuclear export (SINE) compounds reduce cell viability and downregulate XPO1 at the protein level. (**A**) SINE used within this study. (**B**) Cell viability assays with SNU-1 (top panel) and SNU-16 (bottom panel) after 72 h treatment with SINE, positive control (Leptomycin B, LMB) or negative control (KPT-301) where *n* = 3 for both cell lines. All treatment cell counts were normalized to control cell counts. (**C**) Western blot of time course with SNU-16 cell line after treatment with KPT-330, KPT-8602 or KPT-301 at lower and higher doses (*n* = 3). Western blot was probed for XPO1 and beta-actin and densitometry analysis was performed using ImageJ and is representative of three independent experiments.

XPO1 inhibitors used within this study are referred to as selective inhibitor of nuclear export (SINE) compounds (structures given in Figure 2A). The diffuse SNU-1 and SNU-16 cell lines showed remarkable sensitivities to both the first and second-generation SINE (Figure 2B) with IC_50_ values in the low nanomolar range. We then performed a time course experiment to assess the effect of XPO1 protein expression after inhibition with SINE. We found a reduction of XPO1 protein (Figure 2C) as early as 24 h, but was more prominently seen at 48 and 72 h, which is aligned to studies published in other distinct tumor models [25]. 

### 2.3. SINE Interfere with Processes Necessary for Gastric Cancer Cell Survival

**Figure 3:**
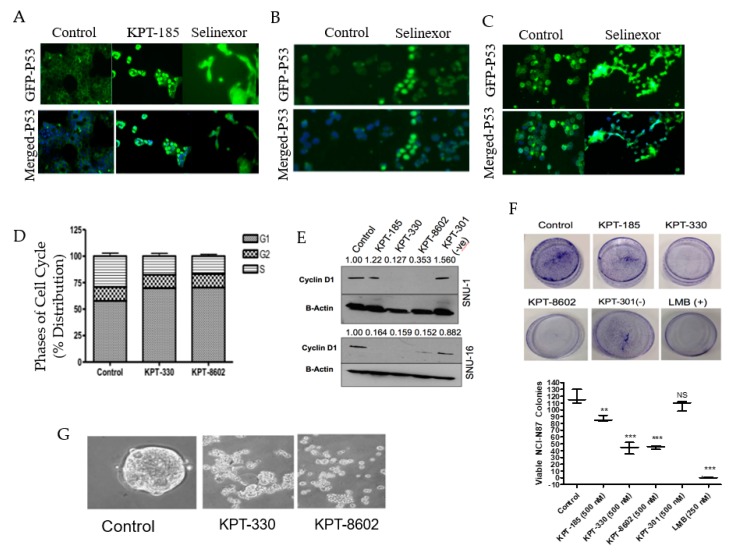
SINE perturb the cell cycle, colony formation, tumor suppressor localization and growth of 3D culture. Immunofluorescence analysis at 2 h after KPT-185 or KPT-330 treatment for nuclear localization of p53 in (**A**) NCI-N87 (2 μM), (**B**) SNU-1 (150 nM) and (**C**) SNU-16 (150 nM) cell lines. (**D**) Cell cycle analysis of NCI-N87 after 72 h treatment with KPT-330 (1 μM) and KPT-8602 (1 μM) (*n* = 3). (**E**) Molecular analysis of the SNU-1 and SNU-16 cell lines after 72 h treatment with KPT-330 (150 nM), KPT-8602 (150 nM), KPT-185 (150 nM) and KPT-301 (150 nM). Blots were probed for cyclin D1 and beta-actin (*n* = 3). Densitometry analysis was provided using ImageJ and is representative of three independent experiments. (**F**) Colony formation assay of NCI-N87 cell lines treated with SINE (500 nM), LMB (+ control, 250 nM) or KPT-301 (− control, 500 nM). Quantification is provided below and is representative of three independent experiments. (**G**) Spheroid formation assay with NCI-N87 cell line grown in spheroid formation media (see Section 4). Cells were treated with 500 nM KPT-330 or KPT-8602 for 10 days and imaged using microscopy. ** *p* < 0.01, *** *p* < 0.001, NS: Not significant.

As discussed previously, the primary function of XPO1 is to export proteins and mRNA from the nucleus into the cytosol. Therefore, we evaluated whether SINE treatment induced nuclear retention of TSPs. We looked at the localization of well-known tumor suppressor protein TP53 after SINE treatment in the NCI-N87, SNU-1 and SNU-16 cell lines (Figure 3A−C) and observed substantial nuclear retention compared to the control. Earlier studies have shown that the naturally occurring and irreversible XPO1 inhibitor Leptomycin B (LMB) inhibits cell cycle progression from the G1/S phase [26]. We examined whether treatment with SINE also inhibited cell cycle progression in our gastric models. After treatment with KPT-330 or KPT-8602 we observed a reduction in the S phase and enhancement of the G1 phase (Figure 3D) in the NCI-N87 cell line. Furthermore, we identified significant downregulation of cyclin D1, a prominent G1 progression protein (Figure 3E) in the SNU-1 and SNU-16 cell lines. To identify whether SINE can reduce colony formation, we utilized the adherent NCI-N87 cell line and treated with SINE, LMB (positive control) or KPT-301 (negative control). Our results demonstrate colony formation is inhibited by the presence of KPT-330 and KPT-8602 (Figure 3F). These results are confirmed by the positive (KPT-185, LMB) and negative controls (KPT-301) (Figure 3F). We also tested the impact of SINE on 3D culture models of gastric cancer. After 10 days of treatment with SINE, there was a significant disruption of spheroids derived from NCI-N87 gastric cancer cell lines (Figure 3G). Collectively, our results strongly demonstrate the impact of SINE on gastric cancer proliferation in 2D and 3D cultures.

### 2.4. SINE Induce Apoptosis in Gastric Cancer Cell Lines

**Figure 4:**
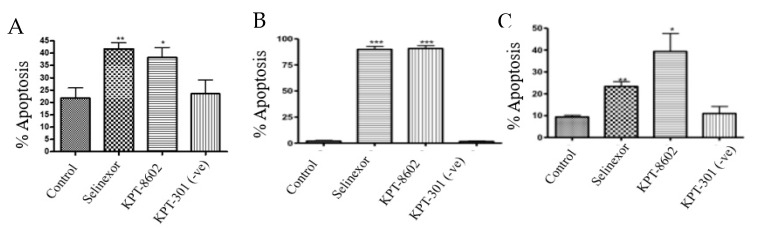
SINE induce apoptosis. (**A**) NCI-N87 cells were treated with 1 μM KPT-330, KPT-8602 or KPT-301 for 72 h and analyzed for cellular death using flow cytometry (*n* = 3). * *p* < 0.05, ** *p* < 0.01. (**B**) SNU-1 cells were treated with 300 nM KPT-330, KPT-8602 or KPT-301 for 72 h and analyzed for cellular death using flow cytometry (*n* = 3). *** *p* < 0.001. (**C**) SNU-16 cells were treated with 300 nM KPT-330, KPT-8602 or KPT-301 for 72 h and analyzed for cellular death using flow cytometry (*n* = 3). * *p* < 0.05, ** *p* < 0.01.

To determine whether inhibition of XPO1 leads to an induction of cellular death, we performed Annexin V FITC and 7-aminoactinomycin D (7AAD) apoptotic assay. Exposure to SINE compounds for 72 h resulted in significant apoptotic induction in NCI-N87, SNU-1 and SNU-16 cells (Figure 4A–C).

### 2.5. XPO1 Inhibition Causes Perturbation of Small RNA Expression

**Figure 5:**
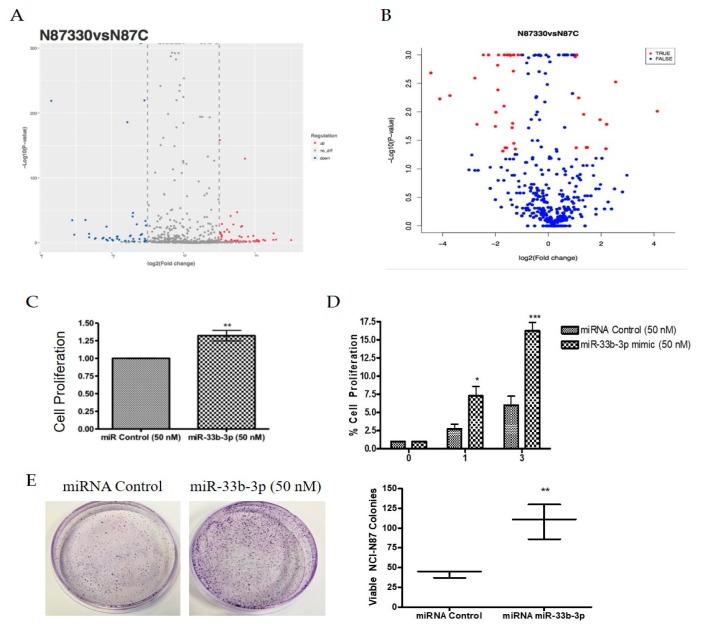
SINE alter non-coding RNA expression. (**A**) MiRNA differential expression represented by a volcano plot in NCI-N87 cells after 48 h treatment with 500 nM KPT-330. (**B**) PiRNA differential expression represented by a volcano plot in NCI-N87 cells after 48 h treatment with 500 nM KPT-330. (**C**) Growth analysis after 24 h incubation with 50 nM miR-33b-3p mimic using 3-(4,5-dimethylthiazol-2-yl)-2,5-diphenyltetrazolium bromide Assay (MTT) growth assay (*n* = 3). ** *p* < 0.01. (**D**) Time course growth analysis using cell viability assay in SNU-1 cell line after 24 h incubation with 50 nM miR-33b-3p mimic where * is representative of * *p* < 0.05, ** *p* < 0.01 and *** *p* < 0.001 (*n* = 3). * *p* < 0.05, *** *p* < 0.001. (**E**) Colony formation assay in NCI-N87 cell line after double transfection (72 h each) of 50 nM miR-33b-3p mimic or miRNA control. Cells were plated at low density (1000 cells/dish), grown in regular media and stained with Coomassie blue for visualization (*n* = 3). Quantification is provided and is representative of three independent experiments. ** *p* < 0.01.

MicroRNAs and other mRNAs are normally exported from the nucleus by XPO5. However, studies have shown that aside from exporting TSPs, XPO1 can also transport certain mRNAs and non-coding RNAs to the cytosol [27,28,29,30,31]. In earlier experiments, we demonstrated that XPO1 inhibition could inhibit oncogenic microRNAs and simultaneously upregulate tumor suppressor microRNAs in pancreatic ductal adenocarcinoma models [32]. Several non-coding RNAs, particularly piRNAs, have been implicated in the biology of gastric cancer [28] and more recently, piRNA biogenesis has been linked to XPO1 activity in a *Drosophila* model [33]. We sought to evaluate whether XPO1 inhibition could affect small noncoding RNA expression in gastric cancer models. To address this, we performed non-coding RNA sequencing in the NCI-N87 cell line after treatment with either KPT-330 or KPT-8602. SINE treatment resulted in statistically significant differential expression of various miRNAs and piRNAs compared to the control (Figure 5A,B; Table 1 and Table 2). These results suggest that XPO1 inhibition may alter various tumor suppressive or oncogenic small non-coding RNAs that may play a key role in gastric cancer pathogenesis. We have begun to characterize the miRNAs identified through sequencing by prioritizing our differentially expressed miRNAs based on relevance to gastric cancer. In doing so, we found that certain miRNAs are candidates for involvement in gastric cancer survival, including miR-33b-3p. Utilizing a miR-33b-3p mimic, we found that miR-33b-3p significantly enhanced cellular growth in the NCI-N87 and SNU-1 cell lines (Figure 5C,D) as well as influenced colony formation (Figure 5E) in the NCI-N87 cell line suggesting an oncogenic role in gastric cancer.

### 2.6. KPT-330 Exhibits Synergy with Paclitaxel In Vitro and In Vivo

**Figure 6:**
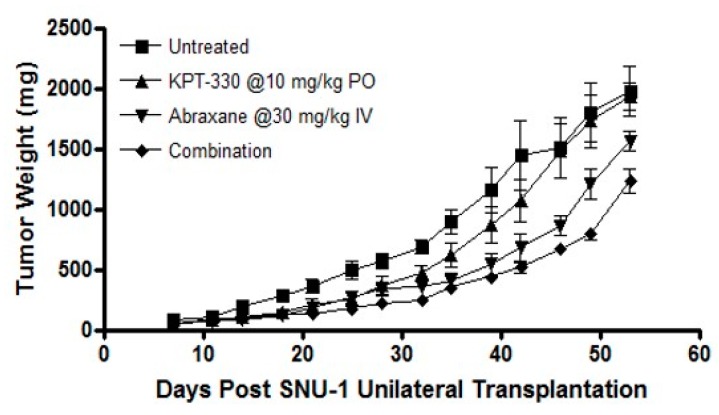
In vivo SINE study. In vivo analysis of KPT-330 and paclitaxel combination in ICR-nude female mice with subcutaneous implantation of SNU-1 xenograft.

XPO1 inhibition has been shown to synergize with several chemotherapeutics, small molecular drugs, radiation therapies and immune checkpoint inhibitors [34,35]. Encouraged by these published findings we further evaluated whether SINE compounds could enhance the efficacy of a standard of care drug paclitaxel that is used clinically in gastric cancer. For these studies we combined various doses of selinexor or eltanexor with paclitaxel and observed the synergy (Combination Index (CI) <1) (Table 3). We then preformed in vivo analysis with single agent KPT-330 or a combination with nab-paclitaxel. Oral administration of selinexor at a sub-optimal dose of 10 mg/kg every other day for three weeks had minimal anti-tumor activity. Similar results were observed with nab-paclitaxel given at 30 mg/kg weekly for three weeks. However, the combination of selinexor and nab-paclitaxel resulted in statistically significant enhanced tumor inhibition compared to single agent treatments (Figure 6) in the SNU-1 xenograft suggesting that selinexor can be used in conjunction with standard of care chemotherapeutics such as nab-paclitaxel.

## 3. Discussion

In this article we demonstrate the therapeutic potential of targeting the nuclear export protein exportin 1/ chromosome region maintenance 1 (XPO1/CRM1) in gastric cancer. We show that targeted inhibition of XPO1 can realign several non-coding RNAs that could also impact gastric cancer cell growth. These observations prove that (1) XPO1 is a valid therapeutic target in gastric cancer and we (2) lay the foundation for the identification of future biomarkers (non-coding RNA) that can be used to stratify patients who would best respond to SINE therapy. These studies strengthen the rationale of bringing SINE into the clinic for gastric cancer. 

Gastric cancer remains a deadly disease and its incidence is rising. Many causative factors have been identified for gastric cancer including diet, obesity, Epstein Barr virus (EBV), genetic predisposition as well as infection of the carcinogenic bacteria *H. pylori* [36,37,38]. For *H. pylori*-positive disease, controlling the infection has been proposed as a possible therapeutic avenue [39]. However, eradication of the bacteria or creating an EBV vaccine [40] may not fully prevent gastric cancer due to diet, lifestyle and those with a genetic component including the BRCA mutation, CDH1 deletion or Lynch syndrome [41,42,43,44]. These patients may face the option of either partial or complete stomach removal for prevention, which contributes to a decreased quality of life [45]. There have been suggestions to modify the current standard of care guidelines, as was derived from the FLOT4 clinical trial, which showed encouraging results using a combination treatment of docetaxel, Oxaliplatin and fluorouracil/leucovorin, but the overall survival increased only 15 months [46]. Gastric cancers occur most frequently in the elderly and many of these patients have poor health making them ineligible for chemotherapeutic combinations that have harsh associated side effects. Clearly, there is an urgent need for development of newer and more efficacious molecular-based treatments.

Aberrations in the nuclear transport machinery have been reported in several malignancies [10,11,12]. Cells rely on the importins and exportins shuttles to move various proteins and RNAs in and out of the nucleus in a tightly controlled fashion. This in turn assists in the precise compartmentalization of tumor suppressors and genome surveillance proteins in the correct cellular compartment of the cell [8,9]. There is ample evidence to suggest that pronounced expulsion of nuclear tumor suppressor and genome surveillance proteins to the cytosol by XPO1 leads to TSP inactivation through mislocalization [10,11,12]. These observations give traction to the concept of targeting the nuclear export machinery as a therapeutic strategy for cancer [13,14,15,16,17,18].

Our analysis of clinical gastric samples showed overexpression of XPO1 in cancer cells compared to normal tissue and established relevance and rationale for targeting XPO1 in patients. We demonstrated strong anti-tumor activity of SINE compounds as single agents and in combination with standard chemotherapies for gastric cancer in vitro and in vivo. Aside from selinexor, we have also demonstrated that the second-generation SINE compound KPT-8602 is just as effective in reducing gastric cancer growth while exhibiting fewer side effects, all of which corresponds with existing published studies [47,48]. There are recent studies aimed at determining the mechanism of the action of selinexor. Data from a recent publication suggested that upregulation of TP53 could be a possible mechanism [49]. However, previous publications have demonstrated effectiveness of SINE compounds regardless of p53 status, function or regulatory changes in distinct tumor models [18]. More work is needed in order to pinpoint the major TSPs and additional players responsible for the observed anti-tumor action by SINE.

Alterations in small RNA expression have recently been characterized in gastric tumors [27,28,50]. Small RNAs including piRNAs, lncRNAs and miRNAs are known to regulate several cellular functions related to gastric cancer survival [51,52]. In our proof-of-principle study, for the first time, we demonstrate that SINE compounds can globally alter miRNA expression in gastric cancer models and uncover novel miRNAs that are differentially expressed following SINE treatment. Uncovering the underlying function of miRNAs and piRNAs may prove to be of prognostic value and may allow for a better selection of patients that would respond beneficially to SINE compound treatment. For a rationale as to why paclitaxel would be a sufficient drug to combine with SINE, we utilized the Oncomine database and compared the genetic alterations in a gastric cancer paclitaxel-sensitive cell line to a normal gastric cancer cell line. We found a handful of genes that were altered after paclitaxel treatment including nuclear transport proteins like XPO7. XPO7 is part of the nuclear export family that is not well studied. However, XPO7 knockdown has been implicated as a probable therapeutic target in conjunction with XPO1 knockdown in various neurodegenerative diseases such as dementia, amyotrophic lateral sclerosis (AML) and ovarian cancer and its overexpression in cancer results in poor outcomes [53,54]. Although KPT-330 is specifically known to target XPO1, we hypothesized that combination treatment of paclitaxel with SINE may perturb additional members of the nuclear transport system to allow for a more enhanced therapeutic effect on gastric cancer cells. Combining KPT-330 with paclitaxel exhibited synergy both in vitro and in vivo. We hypothesize that this favorable drug interaction may also be due to the combined G1/S and G2/M phase arrests, in which G2/M phase arrest is consistent with published literature on paclitaxel [55]. Another explanation might otherwise be due to the synergy between inhibited microtubules due to paclitaxel and inhibited centromere formation via XPO1 inhibition [56]. The mechanism underlying the observed synergistic interaction between these two compounds is currently ongoing and beyond the scope of this manuscript.

**Figure 7:**
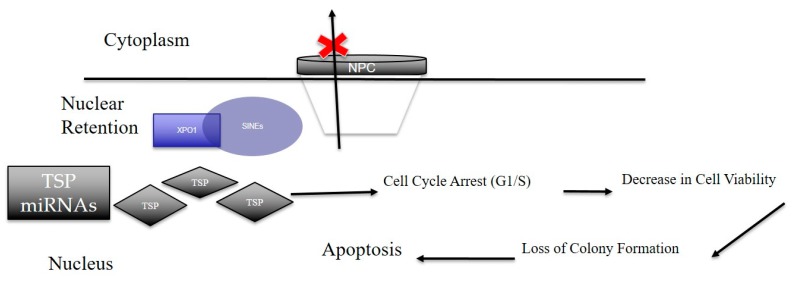
Overall scheme of how SINE elicit anti-tumor effects in gastric cancer. Nuclear retention of XPO1 via SINE results in the global suppression of nuclear export of tumor suppressor proteins (TSPs) and tumor suppressor microRNAs. Once arrested, these TSPs elicit functions such as cell cycle arrest, decrease in cell viability and loss of colony formation. This disruption in normal cancer cellular processes leads to cellular death resulting in an anti-cancer effect. Black Arrows are indicative of cellular processes driven by tumor suppressor proteins when retained in the nucleus via suppression of XPO1 (indicative by the red X).

In conclusion, our results strongly point to the critical role of XPO1 in gastric cancer subsistence Figure 7). The observed inhibitory effect of clinical SINE compounds against gastric models in vitro and in vivo strengthens the idea of using XPO1 inhibitors for the treatment of this therapy-resistant disease.

## 4. Materials and Methods

### 4.1. Cell Lines, Culture Conditions and Reagents

NCI-N87, SNU-1 and SNU-16 cells were purchased from American Type Culture Collection (ATCC, Manassas, VA, USA) and were maintained in RPMI-1640 (Invitrogen, Carlsbad, CA, USA) supplemented with 10% fetal bovine serum (FBS) and 1% penicillin/streptomycin (P/S) in 5% CO2 atmosphere at 37 °C (referred to as complete medium). All cells were used up to 25 passages. Cell lines were purchased in 2017 and were tested and authenticated most recently on August 28, 2018. Primary antibody against XPO1 was purchased from Santa Cruz Biotechnologies (Dallas, TX, USA). Cyclin D1 and p53 antibodies were purchased from Cell Signaling Technologies (Danvers, MA, USA). Beta-actin was purchased from Sigma (St. Louis, MO, USA). Anti-Rabbit Secondary Antibody was purchased from Santa Cruz Technologies (Dallas, TX, USA) and Anti-Mouse Secondary Antibody was purchased from Thermo Fisher (Waltham, MA, USA). Annexin V FITC apoptosis kit was purchased from Biovisions (San Francisco, CA, USA) and 7AAD was purchased from Thermo Fischer Scientific (Waltham, MA, USA).

### 4.2. Annexin V and 7-Aminoactinomycin D (7AAD) Staining Assay

NCI-N87 cells were seeded at a density of 5 × 10^5^ cells/mL. The subsequent day SINE compounds were added at indicated concentrations taken from 1 mM stock in DMSO. After 72 h of incubation, the cells were assessed using Annexin V Reagent Kit according to manufacturer’s protocol (BD Biosciences, San Jose, CA, USA). SNU-16 and SNU-1 cells were seeded at a density of 5 × 10^5^ cells/mL. The following day, SINE compounds were added at the indicated concentration taken from 1 mM stock in DMSO. After 72 h of incubation, the cells were assessed using 7AAD reagent according to the manufacturer’s protocol. All cells were subjected to flow cytometry via the Flow Cytometry Core Facility at Karmanos Cancer Institute using the BSL-II flow cytometer.

### 4.3. Cell Cycle Arrest

NCI-N87 cells were plated at a density of 5 × 10^5^ cells/mL in RPMI without FBS. The following day fresh complete media along with SINE was added to the cells at the indicated concentration. Cells were incubated for 72 h then collected and fixed with 70% cold ethanol. Propidium iodide (stock 1 mg/mL) and RNAse I was then added to the cells, incubated and submitted to the flow cytometry core at Karmanos Cancer Institute for processing using the BSL-II flow cytometer.

### 4.4. Colony Formation

NCI-N87 cells were plated at a density of 75% confluency in complete medium. The following day fresh complete media along with SINE was added to the cells at the indicated concentration. Cells were incubated for 72 h, trypsinized and collected. After collection, cells were plated at very low density (1 × 10^3^ cells/mL) in complete media and incubated until confluency was reached in any of the plates. Plates were then fixed with 100% methanol for 5 min and stained with Coomassie blue.

### 4.5. Growth Inhibition by 3-(4,5-Dimethylthiazol-2-yl)-2,5-diphenyltetrazolium Bromide Assay (MTT)

NCI-N87 cells were seeded at a density of 1 × 10^4^ cells per well in 96-well micro-titer plate in complete media. The following day the medium was removed and replaced with medium containing SINE compounds at the indicated concentrations (0−500 nM) taken from 1 mM stock dissolved in DMSO. After 72 h of incubation, the MTT assay was performed by adding 20 µL of 3-(4,5-dimethylthiazol-2-yl)-2,5-diphenyltetrazolium bromide (MTT) (Sigma, St. Louis, MO, USA) solution (0.5 mg/mL in PBS) to each well and incubated for 2 h. After the supernatant was aspirated, cells were dissolved in 100 µL of 100% 2-propanol. The plates were rocked for 30 min in a gyratory shaker and absorbance was measured at 570 nm via a plate reader.

### 4.6. Growth Inhibition using Trypan Blue Cell Viability Assay

Suspension SNU-1 and SNU-16 cells were seeded at a density of 2.5 × 10^4^ cells/well in a 24-well plate in complete medium. After subsequent acclimation (3−5 h) single agent SINE, Taxol or SINE plus Taxol were added to the medium at the indicated concentrations. SINE compounds or nab-paclitaxel was diluted from 1 mM stock dissolved in DMSO to 1:100 in PBS. After 72 h of incubation, the cells were mixed well with a pipette, collected and diluted 1:1 with Trypan blue, 20 µL each. The cells were subjected to counting via a hemocytometer with a fitted plate.

### 4.7. Immunofluorescence Assay

NCI-N87 cells were grown on glass chamber slides overnight to a confluency of ~50% and exposed to SINE compounds at 2 μM for 2 h. SNU-1 and SNU-16 cells were treated for 2 h with SINE then spun down on a microscope slide using a CytoSpin. At the end of treatment cells were fixed with 4% paraformaldehyde for 10 min (NCI-N87) or 100% cold acetone for 5 min (SNU-1 and SNU-16) and all were permeabilized with Triton−100x. The fixed slides were then blocked in 5% Bovine Serum Albumin (BSA) and probed with primary and secondary antibodies according to Cell Signaling Immunofluorescence General Protocol in 3% BSA. The slides were dried and ProLong Antifade mounting medium (Thermo Fisher, Waltham, MA, USA) was added under the coverslip. The slides were analyzed under an inverted fluorescent microscope.

### 4.8. In Vivo Model

Under our Wayne State University 2016 approved Institutional Animal Care and Use Committee (IACUC) application, 24 ICR-Scid female mice (Taconic Biosciences, Rensselaer, NY, USA) were unilaterally and subcutaneously transplanted with about 50–70 mg SNU-1 previously established tumor fragments [32]. One week post implantation, all animals were checked for engraftment, once confirmed (20 out of 24), they were randomly assigned to four cohorts each with five mice: untreated, KPT-330 orally treated (10 mg/kg q3d), nab-paclitaxel intravenously treated (30 mg/kg q3d) and their combination. All mice were followed daily and their body weights, tumor weights (LXW^2^/2) and condition were recorded periodically. The experiment was terminated when tumor burden reached 2000 mg or less. IRB Protocol (16-03-059).

### 4.9. miRNA Transfection

NCI-N87, SNU-1 and SNU-16 cells were grown in 24-well plates in RPMI media and FBS for 24 h. The following day 50 nM of miR-33b-3p mimic (Dharmacon, Lafayette, CO, USA), or miRNA control (Dharmacon) were transfected with Lipofectamine 2000 (1 mg/mL) for 24 h in RPMI + 10% FBS. After transfection, cells were collected and assayed for growth using MTT (for NCI-N87) or cell viability (SNU-1 and SNU-16) with Trypan blue counting. For colony formation, cells were transfected with 50 nM of miR-33b-3p for 72 h and transfected again with 50 nM of miR-33b-3p or miRNA control resulting in a double transfection.

### 4.10. RNA Extraction

NCI-N87 and SNU-1 cells were grown in 100 mm dishes and exposed to media and SINE compound: selinexor or eltanexor at 500 nM concentration or 150 nM concentration, respectively for 48 h. Total RNA was extracted using Trizol reagent (Invitrogen, Waltham, MA, USA) following the manufacturer’s procedure. The total RNA quality and quantity were first verified using our plate reader (TECAN, Durham, NC, USA). Then, analysis of the total RNA was performed on Bioanalyzer 2100 (Agilent, CA, USA) with RIN number >7.0. Approximately 1 ug of total RNA was used to prepare a small RNA library according to protocol of TruSeq Small RNA Sample Prep Kits (Illumina, San Diego, CA, USA). Single-end sequencing was preformed 50 bp on an Illumina Hiseq 2500 at LC Sciences (Hangzhou, China) following the vendor’s recommended protocol. Raw reads were subjected to an in-house program, ACGT101-miR (LC Sciences, Houston, Texas, USA) to remove adapter dimers, junk, low complexity, common RNA families (rRNA, tRNA, snRNA, snoRNA) and repeats. For piRNA the unique sequences with length in 25~37 nucleotides were mapped to specific-species precursors in the piRNA database (Available online: htpp://pirnabank.ibab.ac.in/request.html, accessed on 5 July 2018) by BLAST search to identify known piRNAs, and one mismatch inside of the sequences was allowed in the alignment. The unique sequences mapping to both the piRNA database and species genome were identified as known piRNAs. The unique sequences only mapping to the genome were considered to be piRNA candidates.

### 4.11. Non-Coding RNA Data Analysis (miRNA)

Subsequently, unique sequences with length in 18~26 nucleotides were mapped to specific species precursors in miRBase 22.0 by BLAST search to identify known miRNAs and novel 3p- and 5p-derived miRNAs. Length variation at both 3’ and 5’ ends and one mismatch inside of the sequence were allowed in the alignment. Unique sequences mapping to specific-species mature miRNAs in hairpin arms were identified as known miRNAs. The unique sequences mapping to the other arm of known specific-species precursor hairpin opposite to the annotated mature miRNA-containing arm were considered to be novel 5p- or 3p-derived miRNA candidates. The remaining sequences were mapped to other selected species precursors (with the exclusion of specific species) in miRBase 22.0 by BLAST search, and the mapped pre-miRNAs were further BLASTed against the specific species genomes to determine their genomic locations. The above two we defined as known miRNAs. The unmapped sequences were BLASTed against the specific genomes, and the hairpin RNA structures containing sequences were predicated from the flank 80 nt sequences using RNAfold software (Available online: http://rna.tbi.univie.ac.at/cgi-bin/RNAWebSuite/RNAfold.cgi, accessed on 5 July 2018. The criteria for secondary structure prediction were: (1) number of nucleotides in one bulge in stem (≤12); (2) number of base pairs in the stem region of the predicted hairpin (≥16); (3) cutoff of free energy (kCal/mol ≤−15); (4) length of hairpin (up and down stems + terminal loop ≥50); (5) length of hairpin loop (≤20); (6) number of nucleotides in one bulge in mature region (≤8); (7) number of biased errors in one bulge in mature region (≤4); (8) number of biased bulges in mature region (≤2); (9) number of errors in mature region (≤7); (10) number of base pairs in the mature region of the predicted hairpin (≥12); and (11) percent of mature region in stem (≥80).

### 4.12. Non-Coding Data Analysis (piRNA)

piRNA differential expression based on normalized deep-sequencing counts was analyzed by selectively using the Fisher exact test, Chi-squared 2 × 2 test, Chi-squared nXn test, Student’s *t*-test and ANOVA based on the experiment design. The significance threshold was set to be 0.01 and 0.05 in each test. To predict the genes targeted by differentially expressed piRNAs, two computational target prediction algorithms (Target Scan 50 and miRanda 3.3a) were used to identify piRNA binding sites. Finally, the data predicted by both algorithms were combined and the overlaps were calculated, the Gene Ontology (GO) terms and Kyoto Encyclopedia of Genes and Genomes (KEGG) pathway of these differentially expressed piRNA targets were also annotated.

### 4.13. siRNA Interference

A total of 5 × 10^4^ NCI-N87 cells were plated in 24-well plates with RPMI only and incubated for 24 h. The following day siXPO1 or siControl (10 μM) were mixed with Lipofectamine (1 mg/mL) according to the manufacturer’s protocol and added individually to the cells in medium containing RPMI + 10% FBS. Cells were incubated for 72 h with siRNA/Lipofectamine complexes then trypsinized and were either replated for MTT assay or underwent protein collection.

### 4.14. The Human Protein Atlas Database Search

We utilized the Human Protein Atlas, version 88.38, to obtain survival curves for XPO1 expression. Using this database, we searched for XPO1 and data relevant to gastric cancer. To categorize the data based on gender or stage of disease, we chose the correct annotation and used the suggested cut-off provided and *p*-values were calculated with this program (Available online: https://www.proteinatlas.org/, accessed on 15 August 2019.

### 4.15. Western Blotting

A total of 5 × 10^4^ NCI-N87 XPO1 silenced XPO1 or silenced control cells were collected directly after double transfection with siRNA (see siRNA interference). Then 1 × 10^6^ SNU-1 and SNU-16 cells were plated in complete medium and treated with 150 nM SINE (for cyclin D1 blotting) and SNU-16 cells were also treated with 50 and 100 nM of KPT-330, KPT-8602 or KPT-301 made from 1 mM stock of drug dissolved in DMSO and further diluted 1:100 in PBS. Cells were incubated for the indicated time, collected and lysed using RIPA lysis buffer with protease inhibitors. Cellular lysates were prepared, clarified and protein concentration (μg) was determined using bicinchoninic acid (BCA) protein assay. Proteins were run using SDS-PAGE gel transferred to Polyvinylidene fluoride (PVDF) membrane. All primary antibodies were incubated overnight (1:2500) in 3% milk at 4 °C and secondary antibodies were incubated (1:5000) in 3% milk for 1 h real time (RT) both with intermediate washes with PBS + Tween-20 (0.1%) 5 min each, three times.

### 4.16. 3D Spheroid Culture

NCI-N87 cells maintained in RPMI-1640, 10% FBS and 1% P/S were trypsinized and collected. Cells were resuspended in spheroid growth medium [57] in 1 × 10^3^ cells per well in a 6-well low attachment plate using a cell strainer to get single cell suspensions (Corning, Durham, NC, USA). Media was supplemented every 4 days and spheroid growth was monitored. For treatment, spheroids were transferred to a 24-well low attachment plate (Corning, Durham, NC, USA) in spheroid culture media treated the following day. KPT-330 and KPT-8602 were taken individually from 1 mM stock dissolved in DMSO and treated at 500 nM. IC50 values were found for KPT-330 and KPT-8602 in 2D culture separately. KPT-330 IC50 was 800 nM and KPT-8602 was 500 nM. We wanted to use the same lower concentration of both drugs with the rationale being cells in 3D culture behave differently than in a 2D culture and found a response at this lower concentration. Growth was monitored for 10 days and images were collected by microscopy.

### 4.17. ImageJ Analysis

ImageJ (developed by the National Institute of Health (NIH)) was utilized to perform densitometry analysis. The original Western blots were uploaded to ImageJ software) where the background was subtracted, the image inverted, and the bands were detected by measuring median grey scale. XPO1 or Cyclin D1 values were normalized to their respective beta-actin bands. Values indicated on the blots are representative of the average of three independent experiments (*n* = 3).

### 4.18. Statistical Analysis

Statistical analysis was provided by GraphPad PRISM software (San Diego, CA, USA).

## Figures and Tables

**Table 1:** The top 10 differentially expressed miRNAs that were statistically significant are represented within the table.

microRNA	Expression	Log2 Fold Change
Hsa-miR-130a-3p	Downregulated	−2.16
Hsa-miR-7974	Downregulated	−1.97
Hsa-let-7c-3p	Downregulated	−1.95
Hsa-miR-33b-3p_R-1	Downregulated	−1.89
Hsa-miR-12135_1ss9TC	Downregulated	−1.80
Hsa-miR-409-5p	Upregulated	2.05
Hsa-miR-129-1-3p	Upregulated	2.09
Hsa-miR-548az-5p_L-1R+1	Upregulated	2.18
Hsa-miR-147b-5p	Upregulated	2.24
Hsa-miR-376c-3p	Upregulated	2.96

**Table 2:** The top 10 differentially expressed piRNAs that were statistically significant are represented within the table.

piRNA	Expression	Log2 Fold Change
piRNA-88	Downregulated	−2.78
piRNA-57	Downregulated	−2.70
piRNA-76	Downregulated	−2.45
piRNA-78	Downregulated	−2.00
piRNA-77	Downregulated	−1.91
piRNA-109	Upregulated	1.34
piRNA-20	Upregulated	1.96
piRNA-111	Upregulated	2.55
piRNA-112	Upregulated	4.13

**Table 3:** Synergy analysis of combination paclitaxel and KPT-330 in SNU-1 cell line.

Paclitaxel (nM)	KPT-330 (nM)	Fa Value	Combination Index (CI) Value
12.5	25	0.822	0.486
25	0	0.916	0.530
37.5	75	0.957	0.638
50	100	0.979	0.567

## Abbreviations

XPO1	Exportin 1
miRNA	microRNAs
piRNA	piwiRNA
SINE	Selective inhibitors of nuclear export
